# The Pharmacological Effect of Hemin in Inflammatory-Related Diseases: A Systematic Review

**DOI:** 10.3390/biomedicines12040898

**Published:** 2024-04-18

**Authors:** João Estarreja, Gonçalo Caldeira, Inês Silva, Priscila Mendes, Vanessa Mateus

**Affiliations:** 1 H&TRC—Health and Technology Research Center, ESTeSL—Escola Superior de Tecnologia da Saúde, Instituto Politécnico de Lisboa, 1990-096 Lisbon, Portugal; joaoestarreja@edu.ulisboa.pt (J.E.); 2019152@alunos.estesl.ipl.pt (G.C.); ines.silva@estesl.ipl.pt (I.S.); priscila.mendes@estesl.ipl.pt (P.M.); 2 Research Institute for Medicines (iMed.ULisboa), Faculty of Pharmacy, Universidade de Lisboa, Av. Professor Gama Pinto, 1649-003 Lisbon, Portugal

**Keywords:** hemin, inflammation, oxidative stress, nonclinical studies in vivo, animal models

## Abstract

Background: Hemin is clinically used in acute attacks of porphyria; however, recent evidence has also highlighted its capability to stimulate the heme oxygenase enzyme, being associated with cytoprotective, antioxidant, and anti-inflammatory effects. Indeed, current preclinical evidence emphasizes the potential anti-inflammatory role of hemin through its use in animal models of disease. Nevertheless, there is no consensus about the underlying mechanism(s) and the most optimal therapeutic regimens. Therefore, this review aims to summarize, analyze, and discuss the current preclinical evidence concerning the pharmacological effect of hemin. Methods: Following the application of the search expression and the retrieval of the articles, only nonclinical studies in vivo written in English were considered, where the potential anti-inflammatory effect of hemin was evaluated. Results: Forty-nine articles were included according to the eligibility criteria established. The results obtained show the preference of using 30 to 50 mg/kg of hemin, administered intraperitoneally, in both acute and chronic contexts. This drug demonstrates significant anti-inflammatory and antioxidant activities considering its capacity for reducing the expression of proinflammatory and oxidative markers. Conclusions: This review highlighted the significant anti-inflammatory and antioxidant effects of hemin, providing a clearer vision for the medical community about the use of this drug in several human diseases.

## 1. Introduction

**Figure 1:**
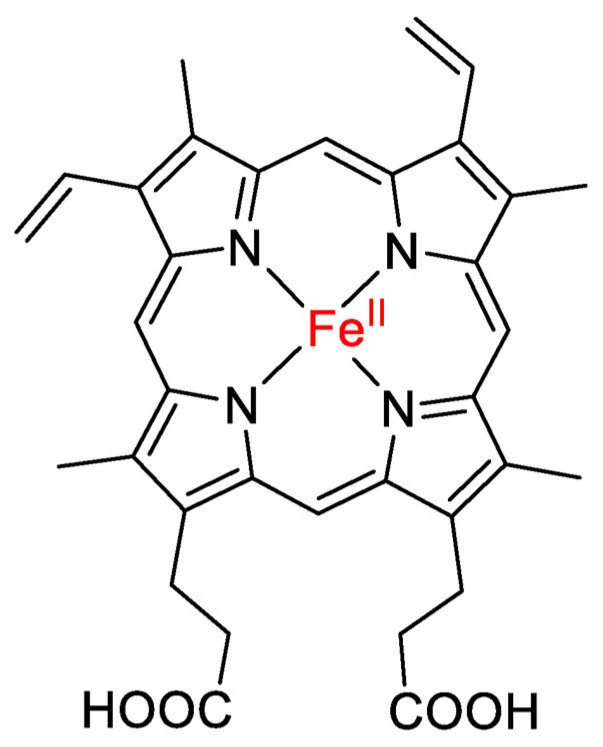
Structure of hemin (adapted from Tahoun et al., 2023) [6].

Hemin, also known as ferriprotoporphyrin IX chloride (Figure 1), is an iron-containing metalloporphyrin, commonly used in the treatment of acute attacks of porphyria [1,2]. Theoretically, porphyria is intimately related to a deficient heme biosynthesis pathway, resulting in a lack of heme necessary to produce several hemoproteins. Additionally, it can also result in the accumulation of heme precursors, which are directly and indirectly toxic to the human organism [2,3,4]. Hemin reduces heme deficiency by suppressing delta-aminolaevulinic acid synthase activity. Consecutively, it decreases the concentration of porphyrins and toxic precursors of heme [2,4,5]. In this sense, once the levels of hemoproteins and respiratory pigments become normal and stable, the biological abnormalities observed in patients with porphyria are attenuated [4,5].

Recent evidence has demonstrated that hemin also has the capability of inducing the expression of the heme oxygenase (HO) enzyme, known as a rate-limiting enzyme for heme catabolism, responsible for producing biliverdin, free iron, and carbon monoxide [7,8,9]. Currently, there are three HO isoenzymes identified in mammals, namely HO-1, HO-2, and HO-3, where the first one is usually associated with cytoprotective, anti-inflammatory, and antioxidant activities [7,8,10,11]. Importantly, the induction in HO-1 is intimately related to the capability of hemin to activate the nuclear factor erythroid 2 related factor 2, and, at the same time, to inactivate the repression factor Bach1 [12]. Moreover, preclinical evidence has shown significantly higher values of HO-1 and HO-1 mRNA in inflammatory-related disorders, like, for example, colitis [7,8,11]. The expression of this isoenzyme is usually observed in macrophages; however, it can also be noticed in epithelial cells present in the region where the inflammatory response is occurring [13,14]. Considering the information above, hemin appears as a new potential pharmacological approach in several inflammatory-related human disorders due to its high capacity to stimulate the expression of HO-1.

Currently, an interesting research question for future studies has arisen concerning the existence of several nonclinical studies in vivo that demonstrate the capability of hemin to reduce inflammatory responses. It is essential to understand the underlying mechanism(s) behind the anti-inflammatory and antioxidant effects of this drug to evaluate its potential role in clinical practice. Even though there is already a significant number of nonclinical studies demonstrating the effect of hemin on inflammation, there is an absence of review articles that analyze and criticize the evidence available. In addition, considering the different therapeutic regimens currently described, it is essential to investigate the most optimal ones. Therefore, according to this major gap in the literature, this systematic review aims to summarize, analyze, and discuss the preclinical evidence that is currently available regarding the pharmacological effect of hemin in animal models of disease.

## 2. Materials and Methods

The present systematic review was developed considering the Preferred Reporting Items for Systematic Review and Meta-Analysis (PRISMA 2020) guideline [15]. Additionally, the protocol of this review was registered in the International Prospective Register of Systematic Reviews—PROSPERO (CRD42023406160) and published in an international research journal [16].

### 2.1. Eligibility Criteria

The studies will be selected according to the following inclusion criteria: only original articles; studies where a pharmacological treatment with hemin was evaluated; studies that analyzed inflammation and/or oxidative stress; nonclinical studies in vivo with rodents; and articles only published in English. In addition, regarding the exclusion criteria, the following were considered: review articles; expert opinions; book chapters; studies with only in vitro procedures; and experiments that evaluated the treatment with hemin in diseases present in the summary of product characteristics (e.g., porphyria). 

### 2.2. Information Sources and Search Strategy

The biomedical electronic databases used for this highly sensitive search strategy were MEDLINE (Pubmed), Web of Science, and Scopus. The search was only limited by the publication date, namely between 2015 and March 2023. A comprehensive research expression was developed using descriptors related to three terms (hemin, inflammation, and nonclinical study) and their synonyms combined with Boolean operators “AND” and “OR” to identify and select the eligible studies. The search strategies, adapted for each biomedical electronic database, are available in the Supplemental Material.

### 2.3. Selection Process

Upon the application of the search expression to the respective biomedical electronic databases, the retrieved articles were exported to a Systematic Reviews Web Application (Rayyan QCRI, Rayyan Systems, Cambridge, MA, USA). Following the exclusion of duplicates, the titles and abstracts of the studies were analyzed by two independent reviewers to select the relevant and potentially eligible articles. Afterward, the two independent reviewers assessed the full text of each potentially eligible article, deciding if it was included or not, considering the eligibility criteria established. In these two previous steps, a third element was included in case of discrepancies between the two reviewers to make a final decision. 

### 2.4. Data Collection Process

The data present in the included studies were carefully extracted to the Excel software (Version 2403, Microsoft, Redmond, WA, USA) by the same two independent reviewers. A standardized data extraction document in Excel sheets (Microsoft) was developed to extract the information of interest, which considered article identification (first author’s name and year of publication); hemin-related parameters; disease animal model; and outcomes assessed. All the information of interest was extracted from the text, graphs, and/or tables from each included study.

### 2.5. Data Items

#### 2.5.1. Population

Only rodent models where the data of interest to be extracted were related to species, strain, gender, and age were considered.

#### 2.5.2. Intervention

Only hemin administration where the parameters of interest were related to dosage, frequency of administration, route of administration, and duration of treatment was considered.

#### 2.5.3. Comparator

No comparator parameters were considered.

#### 2.5.4. Outcomes

The data concerning the outcomes were related to inflammatory and biochemical markers (continuous quantitative measure), macroscopical evaluation (dichotomous measure), and microscopical evaluation (dichotomous measure).

#### 2.5.5. Study Design

Throughout the analysis of the nonclinical studies in vivo, the data of interest to be extracted were related to the disease induced, severity, and chronicity.

### 2.6. Quality and Risk of Bias Assessment

To evaluate the internal validity of each study, along with the methodological quality, an analysis of the risk of bias through the application of SYRCLE’s risk of bias tool was considered [17]. For each study, the grading of all components present in SYRCLE’s tool was performed considering the following statements: complete (2 points); incomplete (1 point); or not mentioned (0 points). The final grade was decided concerning the average of all individual components, and the studies were assigned into the following categories of quality: high (15–20 points); moderate (8–14 points); or low (0–7 points). This evaluation was performed by the same two independent reviewers. Any disagreements were discussed and arbitrated by a third reviewer.

## 3. Results

### 3.1. Study Selection

**Figure 2:**
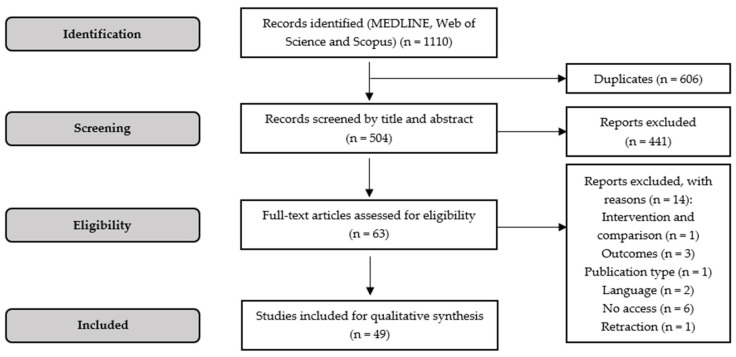
PRISMA flow diagram representing the selection process.

The application of the search strategies, each one in the respective biomedical electronic database, retrieved a total of 1110 articles, where 606 were identified as duplicates (Figure 2). The analysis of the titles and abstracts from the remaining 504 articles resulted in the exclusion of 441 reports. In this sense, the full text of the 63 potentially eligible studies was critically assessed considering the inclusion and exclusion criteria. After this process, 13 articles were excluded concerning the following justifications: a study that applied a combined treatment of hemin with other active pharmacological substance (*n* = 1); studies that did not evaluate inflammatory and oxidative stress biomarkers (*n* = 3); review (*n* = 1); studies written in Chinese (*n* = 2); studies with no access for full-text analysis (*n* = 6), and a publication with retraction order (*n* = 1). The 49 studies included for qualitative synthesis met all the eligibility criteria, in which the potential anti-inflammation of hemin in rodent models of disease was adequately evaluated (Table 1).

### 3.2. Study Characteristics

Upon the inclusion of the 49 eligible studies, an extensive analysis was performed in each one to extract the relevant data, namely hemin- and animal-related parameters, along with the biomarkers assessed in the respective experiments (Table 1). 

### 3.3. Risk of Bias Assessment

In the evaluation of the internal validity and methodological quality of each study, considering the application of SYRCLE’s tool for risk of bias assessment, it was possible to observe an absence of low-quality articles (Table 2 and Table S1 (Supplementary Material)). Indeed, 5 articles reached the highest quality level, while the other 44 were categorized as moderate.

## 4. Discussion

Current preclinical evidence demonstrates a significant anti-inflammatory effect of hemin in different scenarios, such as inflammatory bowel disease [7,8,44,65], kidney dysfunction [23,60], sepsis [40,66,67], arthritis [56,68], pancreatitis [32,53,69], cardiac infarction [46], and airway inflammation [27,29,70]. Considering the long-term toxicity of the current pharmacological treatments applied in chronic inflammatory diseases, it is essential to investigate safer options, either by the development of new molecules or by the process of drug repurposing [71]. Hemin appears as an interesting pharmacological approach in future treatment protocols of chronic inflammatory diseases concerning its efficacy and safety demonstrated in several nonclinical studies in vivo, as previously referred to. Regarding the different treatment protocols applied in preclinical studies, showing high variability in terms of doses, frequency, routes of administration, and duration, it is particularly difficult to understand the most optimal ones. Therefore, it is essential to analyze, summarize, and discuss several therapeutic regimens, providing a well-developed basis for future studies regarding the potential off-label use of hemin in inflammation-related disorders.

### 4.1. Hemin-Related Parameters

#### 4.1.1. Dose

The studies analyzed demonstrated a high variability concerning the dose of hemin administered, where the most commonly used dose was 30 mg/kg (*n* = 7), followed by 50 mg/kg (*n* = 5). Indeed, it is possible to observe a wide range of doses, between 75 µg/kg and 50 mg/kg, which varies considering the route of administration and the animal model chosen. The anti-inflammatory effect of hemin was already possible to observe with a relatively low dose, namely 75 µg/kg, administrated in an animal model of acute pancreatitis, represented by a significant reduction in pathological score in the liver and pancreas, as well as a reduced expression of a proinflammatory cytokine, namely tumor necrosis factor-α (TNF-α) [33]. As expected, when hemin is used at a higher dose, such as 50 mg/kg, significant anti-inflammatory and antioxidant effects are also observed, which translate to reduced concentrations of proinflammatory cytokines (e.g., interleukin (IL)-1β, -6, nuclear factor kappa B (NF-Kb), and TNF-α) and oxidative markers (e.g., malondialdehyde (MDA) and myeloperoxidase (MPO)) [55,56,57,58,59,60]. Importantly, regarding the studies that administered higher doses of hemin, no significant side effects were identified.

In clinical practice, hemin is normally administered at a dose of 3–4 mg/Kg/day in the treatment of acute attacks of porphyria [72]. Therefore, it is possible to consider that this drug achieves its significant pharmacological activity at relatively low doses, concerning both preclinical and clinical settings.

Recently, it was described that HO-1 may influence the enzymatic activity of certain cytochrome P450s (e.g., CYP1A2, CYP2C2, CYP2E1, and CYP3A4). In fact, this can be justified by the fact that HO-1 forms homomeric complexes with these P450s enzymes, affecting their overall activity [73]. Then, the therapeutic regimen (dose, frequency, and duration of treatment) should equate to the potential influence of hemin on the metabolism of other drugs and xenobiotics in general, since it may increase the risk of toxic events.

#### 4.1.2. Frequency of Administration and Duration of Treatment

Regarding the frequency of administration of hemin, it is possible to conclude that the majority of the studies preferred a single-dose treatment (*n* = 22), followed by daily administrations (*n* = 11) and every two days (*n* = 5). Considering the daily treatment, the spectrum of time varies between 2 and 14 days, depending on the disease induced and the chronicity of the therapeutic regimen desired. On the other hand, some studies decided to administer hemin two or three times per week for an extended therapeutic regimen between 8 and 20 weeks. It can be justified by the longer time needed to develop the respective animal model of disease (e.g., emphysema, obesity, steatohepatitis, and liver fibrosis) and the interest in evaluating the potential anti-inflammatory effect of hemin throughout the induction process [18,19,20,50]. Concerning the results obtained in the various studies, it is possible to observe that treatment with hemin demonstrated a significant anti-inflammatory effect in both acute and chronic settings, without showing significant side effects upon its use.

In clinical practice, the standard treatment of acute attacks of porphyria with hemin is through daily intravenous (IV) doses for four consecutive days [72]. However, it can be adapted to each individual, depending on the severity, to a maximum of 14 consecutive daily doses [74].

#### 4.1.3. Route of Administration

Among the different routes of administration considered in the various studies analyzed, the most used was intraperitoneal (IP) (*n* = 37), followed by subcutaneous (SC) (*n* = 5) and topical (*n* = 3). The decision concerning the route of administration should always consider the species of the animal; the substance to be administered; the location where the effect should be noticed; and the total volume [75,76]. Additionally, some studies performed intragastric (*n* = 1) and pleural (*n* = 1) injections to make sure that hemin achieved the maximum therapeutic effect on the respective location, namely the stomach and lungs, with a lower rate of absorption and distribution [34,37].

Concerning the different parenteral routes of administration used in laboratory animals, the most commonly observed are IP, SC, and IV. Currently, considering clinical practice, hemin is only administered intravenously [72,74]. In the specific case of rodent models, the IP route is the most desirable one, demonstrating relative ease to perform, quickness, and minimal stress on the animals [75]. In addition, it presents a faster absorption rate in comparison with the SC route and is also preferable in the case of larger volumes. Regarding chronic treatments, the IP route is also recommended since repetitive IV access can be challenging over time [75,76]. Additionally, IV administration is easier to perform in rats compared with mice due to increased vessel diameter and visibility. Furthermore, this route of administration presents a major advantage compared with the IP and SC routes, namely the systemic delivery of substances, because it bypasses the need for solute absorption [77]. However, the unique study analyzed that performed IV administrations was in mice, more specifically, three daily injections [35]. Interestingly, significant anti-inflammatory and wound-healing effects from hemin in a topical application were also proven, revealing a potential route of administration to be considered in future preclinical and clinical studies [62,63,64]. 

### 4.2. Animal-Related Parameters

#### 4.2.1. Disease Animal Model

Considering the several studies that evaluated the potential anti-inflammatory and antioxidant effects of hemin, it is possible to emphasize high variability in terms of disease animal models. However, the most commonly used were diabetes mellitus (*n* = 4), acute lung injury (ALI) (*n* = 3), colitis (*n* = 3), and sepsis (*n* = 3). In a general mode, it is also important to consider versability regarding treatment with hemin, demonstrating a significant anti-inflammatory role in various contexts, such as lungs (e.g., emphysema, airway inflammation, and asthma), kidneys (e.g., acute kidney injury, nephrotoxicity, and renal inflammation), liver (e.g., liver fibrosis, steatohepatitis, and acute liver failure), pancreas (e.g., acute pancreatitis), and cardiovascular system (e.g., myocardial infarction, vascular dysfunction, and restenosis).

Diabetes mellitus is a highly prevalent and complex metabolic disorder characterized by hyperglycemia, which is a physiologically abnormal condition represented by continued elevated blood glucose levels [78,79,80]. One of the major concerns about the progression of this disorder is related to complications in other organs, such as the kidneys, brain, heart, and skin [78]. The pharmacological activity of hemin has been tested in rodent models of diabetes mellitus to evaluate its efficacy and safety in nephropathy and wound-healing abnormalities [26,62,63,64]. Considering diabetes-induced nephropathy, hemin treatment was shown to have significant anti-inflammatory (e.g., reduction in TNF-α), antiapoptotic (e.g., reduction in caspase-3), and antioxidant (e.g., reduction in MDA, MPO, and nitric oxide) activities. In addition, it was also proved to have a beneficial effect in terms of renal morphology by decreasing the microscopical destruction observed [26]. On the other hand, concerning the diabetes-induced wound-healing abnormalities, the topical treatment with hemin also showed significant anti-inflammatory (e.g., reduction in TNF-α, IL-6, allied with the increase of IL-10) and antioxidant (e.g., increase in superoxide dismutase (SOD) and catalase, as well as the reduction in MDA and glutathione peroxidase) effects. Furthermore, it was possible to notice antiapoptotic (e.g., reduction in caspase-3), coagulating (e.g., extension of prothrombin and thrombin times and reduction in fibrinogen), and angiogenic (e.g., increase in vascular endothelial growth factor) properties from this drug. Finally, an acceleration in the process of wound closure and re-epithelialization was also observed [62,63,64]. It is important to note that the pharmacological activity of hemin was mainly related to the capacity to induce the expression of HO-1 and not to altering serum glucose levels [26,62,63,64].

ALI is normally caused by several pathological conditions, namely sepsis, pneumonia, multiple traumas, pulmonary contusion, or aspiration of dangerous gas [51]. Regarding its pathogenesis, ALI is characterized by neutrophil infiltration, accumulation of inflammatory mediators, and hyaline membrane formation [31,51,81]. This disorder is recognized as a major concern worldwide since it presents a high rate of mortality and the underlying mechanisms are not fully understood yet [31,51]. The treatment with hemin showed significant anti-inflammatory (e.g., reduction in NF-kB, caspase-1, IL-1β, -6, and TNF-α) and antioxidant (e.g., reduction in MDA, MPO, and reactive oxygen species, as well and the increase in SOD) effects. In addition, hemin was also capable of reducing the microscopical abnormalities related to the ALI induction and, consequently, the mortality rate [31,51,52].

Inflammatory bowel diseases are characterized as chronic inflammatory disorders in the gastrointestinal tract, which can be divided into two phenotypes, namely ulcerative colitis and Crohn’s disease [44]. Currently, there is no cure for these disorders, and the pharmacological approaches only aim to induce and maintain the remission phase in patients, rather than resolving the underlying mechanisms(s) [7,8,44,82]. Treatment with hemin was revealed to be a promising pharmacological approach in the future since it presented significant anti-inflammatory (e.g., reduction in cyclooxygenase-2, IL-1β, -6, -12, -18, interferon-γ, NF-kB, and TNF-α, allied with an increase in IL-10) and antioxidant (e.g., increase in SOD and catalase, as well as a reduction in MDA, MPO, and nitric oxide) properties [7,8,44]. Furthermore, treatment with hemin also showed the capability of partially reverting the destruction observed in the colon through histopathological analysis [7,44].

Sepsis is described as a major life-threatening organ dysfunction provoked by an exacerbated and dysregulated systemic host response to infection [61,83]. Considering the extensive organ abnormalities observed during this event, there are several complications to be noticed [40,61]. Indeed, concerning the preclinical evidence demonstrated in this review, the treatment with hemin was evaluated for different complications, such as muscle atrophy and intestinal injury [40,41,61]. Regarding the results obtained in these studies, it was possible to identify, once again, significant anti-inflammatory (e.g., reduction in caspase-1, -11, IL-1β, -6, -8, -18, TNF-α, and toll-like receptor-4) and antioxidant (e.g., reduction in MDA and glutamate dehydrogenase, allied with an increase in SOD) effects from hemin [38,39,60]. In addition, a significant reduction in the mortality rate after the treatment with this drug was also observed [40,41], as well as a partial reversion of the sepsis-induced damage observed in different organs, like the intestine, liver, lungs, kidneys, skeletal muscles, and heart [40,41,61].

#### 4.2.2. Species and Strain

The use of laboratory animals, mainly rodents (e.g., rats and mice), is recognized as one of the central strategies for the study of the etiology and pathogenesis of several diseases, as well as for the evaluation of new potential pharmacological and nonpharmacological treatments [77,84,85]. Considering the results from this review, it is possible to conclude that the most widely used animal was the rat (*n* = 31), followed by mice (*n* = 18). Indeed, these rodent models present several similarities with humans, regarding gene homology, physiology, anatomy, pathological pathways, and immune system [77,86,87,88]. In addition, they also present some advantages, such as reduced breeding and housing costs, easy handling, relatively small size, short life cycle, and gestation time (19 to 21 days) [77,84,85,86]. Concerning statistical relevance, rodent models are relatively cost-effective, allowing the use of adequate sample sizes for further analysis [84,86].

Besides the high similarity between rodent models, a preference for the use of rats was observed, especially in the scenario of cardiovascular diseases. This preference can be justified by the fact that rats present a higher blood volume when compared with mice, promoting an easier and less stressful collection process. In addition, it is possible to observe more evidence regarding cardiovascular physiology and pathology, as well as disease-linked genome sites, in rats [89]. Nevertheless, the choice of using rats or mice is intimately dependent on researchers’ preference and the disease model presented. Generally, there were no differences regarding the pharmacological activity of hemin between the two rodent models previously discussed.

Regarding the different strains used, it is possible to determine that the most used in rats were Wistar (*n*= 17) and Sprague Dawley (*n* = 12), whereas in mice they were C57BL/6 (*n* = 10) and Balb/c (*n* = 6). The use of genetically modified strains in a higher proportion in mice (e.g., HO-1^flox/flox^ (*n* = 1), A2A (*n* = 1) and A2B knockout (*n* = 1), DO11.10 (*n* = 2), and Bas-TRECK (*n* = 1)) when compared with rats was also observed (e.g., Zucker fatty (*n* = 1) and Zucker lean (*n* = 1)).

The decision regarding a specific strain can be relatively difficult and should consider several parameters, such as the objectives of the experiment, the disease animal model, and reproducibility, as well as ethical and financial viability [90]. Theoretically, each strain demonstrates both advantages and disadvantages in comparison with others that are available. Therefore, the final choice should be made after measuring the pros and cons of each strain. Currently, the most commonly used inbred mouse strains in biomedical research are C57BL/6 and BALB/c. Indeed, as inbred strains, each one of them is recognized as being homozygous and genetically stable, presenting consistent phenotypes. Normally, C57BL/6 mice can be useful in several contexts, being recognized as a general multipurpose model. Furthermore, regarding BALB/c mice, they are usually associated with immunology and infectiology research [91,92]. On the other hand, the Wistar and Sprague Dawley strains are the most popular outbred rats used in preclinical research, especially in toxicological and aging studies. The major differences among these species are related to food intake, growth rate, hormonal expression, and tumorigenesis [93].

#### 4.2.3. Gender

The included studies showed a clear preference for using males (*n* = 35), to the detriment of females (*n* = 10). In addition, four studies did not mention the gender chosen. The major reason for this preference is related to the hormonal differences observed between males and females. Indeed, normally, a high variability regarding hormonal levels in females is observed throughout all experiments due to the estrous cycle [86,94]. As an example, increased concentrations of progesterone generally reduce an inflammatory response, which can be a problematic bias in the results from certain studies, especially when a potential anti-inflammatory drug is being tested [95,96]. However, the use of females is essential in several disease models, namely in the context of mastitis and endometrial hyperplasia, as presented above in Table 1 [47,55]. On the other hand, there are also major concerns regarding the use of males. In fact, they are significantly more territorial than females, increasing the overall stress, risk of fighting, and, consequently, mortality rate [97]. Therefore, males should be monitored regularly to prevent cases of severe skin lesions, which can induce a threatening infection with a potent inflammatory response, directly influencing the well-being of the animals and the results obtained [77,86].

#### 4.2.4. Age

Regarding the age of the animals used in the included studies, it was possible to notice that rats and mice were between 1–16 and 5–12 weeks old, respectively. Generally, it can be highlighted that the majority of the studies used rodents aged between 6 and 12 weeks old. Nevertheless, some studies did not mention the exact age of the animals, confirming only that they were adults (*n* = 5).

In normal conditions, only the use of mice and rats beginning at 4 and 5 weeks old should be considered, respectively. In fact, until these ages, rodents have not yet passed the process of weaning and demonstrate an increased dependency on their mothers to survive [86,94]. Only one study used rats aged 1 week old; however, the experiment aimed to evaluate the hepatotoxicity of acetaminophen in neonatal animals. Interestingly, hemin showed significant anti-inflammatory (e.g., reduction in IL-1β, IL-6, and TNF-α) and antioxidant (e.g., reduction in MDA and increase in GSH) effects [57].

Researchers normally prefer rodents aged between 8 and 12 weeks old; however, it is important to consider the normal developmental processes that are ongoing, which can have a significant impact on the results [98]. Indeed, regarding immunology, B- and T-cell responses are usually mature at around 4 and 8 weeks old, respectively [98,99,100]. Furthermore, the production of these two types of cells increases during the first 26 weeks of life [98,101]. On the other hand, considering aged rodents, differences in terms of drug metabolism (e.g., abnormal gene expression of liver enzymes) and a more robust inflammatory response, allied with severe systemic manifestations, when compared with younger animals are normally observed [86,98,102,103]. However, aged rodents appear as useful models in cardiovascular research since they mimic, in most cases, the normal onset and progression of diseases observed in humans (e.g., diabetes *mellitus* and hypertension) [104]. Therefore, considering the information above, age can be recognized as a determinant factor during experimental research and should be adequate for a study’s objectives since it can directly influence the results obtained.

### 4.3. Biomarkers Assessed

Biomarkers have major importance during pharmaceutical discovery, considering both clinical and preclinical settings, since they permit the evaluation of the efficacy and safety of several molecules [105,106]. Considering the studies analyzed, it was possible to notice a high variability of biomarkers assessed. However, concerning inflammation, the most commonly observed markers were TNF-α (*n* = 32), IL-6 (*n* = 22), IL-1β (*n* = 20), and NF-kB (*n* = 10). Furthermore, considering oxidative stress, the most common biomarkers were MDA (*n* = 20), SOD (*n* = 12), glutathione (GSH) (*n* = 12), and MPO (*n* = 11).

Hemin demonstrated a significant anti-inflammatory effect, which can be justified by its capability to reduce the concentration of proinflammatory cytokines (e.g., IL-1β, -6, and TNF-α), as well as to increase the expression of a well-known anti-inflammatory marker, namely IL-10. In addition, this drug was also capable of decreasing the expression of an important inflammatory transcription factor, that is NF-kB. In normal situations, an inflammatory response is associated with oxidative stress, which plays a pivotal role in the onset and progression of several diseases [107]. In this regard, hemin also showed a significant antioxidant effect, demonstrated by a reduction in oxidative markers (e.g., MDA and MPO), allied with an increase in antioxidant mediators (e.g., SOD and GSH). Considering the scenario of animal models of inflammation, the biomarkers of interest can be divided into several classes (e.g., immune-related effectors, cytokines, chemokines, reactive oxygen and nitrogen species, prostaglandins, cyclooxygenases, acute phase proteins, and transcription factors), highlighting a well-balanced pharmacological effect of hemin concerning the positive results demonstrated above [86].

Finally, it is also important to emphasize that the majority of the studies also assessed the expression of HO-1 (*n* = 35), which appears to be a proof of concept for the stimulating effect of hemin in this enzyme. In fact, HO-1 is recognized as a stress protein and metabolic enzyme associated with regulatory effects in cellular and tissue homeostatic responses, modulation of the immune system, and host defense, as well as being a mitigator of inflammation [108]. Theoretically, HO-1 reduces the concentration of free heme, which is known to be involved in various human diseases due to its capability of interfering with several pathological responses (e.g., stimulation of toll-like receptor 4-dependent inflammatory response, membrane lipid peroxidation, and ferroptosis) [108]. Therefore, according to the current literature, it is possible to positively correlate the expression of HO-1, stimulated by the administration of hemin, as well as significant anti-inflammatory and antioxidant activities, which is an interesting pharmacological approach to be considered in future clinical practice.

Despite the evidence demonstrated in this systematic review, it is important to consider a major limitation regarding the methodology, which is the absence of clinical studies. However, at this date, no clinical trials are studying the potential anti-inflammatory effect of hemin, and, in a general mode, it is possible to notice a relatively reduced number of clinical studies that focus on this topic. In this sense, we decided to only include nonclinical studies in vivo since they present the most controlled environment possible. On the other hand, considering the specificity of the outcomes evaluated related to inflammation and oxidative stress, potentially interesting studies that demonstrated other pharmacological activities of hemin, such as its cytoprotective role, were not included. At the same time, it would also be interesting to evaluate the other side of hemin, more specifically, its potential toxic effect in certain tissues, such as in the neurological and gastrointestinal systems, since it has shown to be cytotoxic to cultured mouse astrocytes and colonic cells, respectively [109,110]. Nevertheless, this review can be the first step for future studies evaluating different pharmacological effects of hemin, excluding those already present in the summary of product characteristics.

## 5. Conclusions

This review highlighted an interesting pharmacological effect of hemin in several rodent models of disease, which was based on its significant anti-inflammatory and antioxidant activities. Regarding the use of this drug, most researchers preferred a dose ranging between 30 mg/kg and 50 mg/kg, administered intraperitoneally in most cases. Interestingly, hemin was successfully used in both acute and chronic contexts, demonstrating its pharmacological activity in different therapeutic regimens. In fact, positive results with only a single administration, as well as after several weeks of treatment, were observed. Generally, a significant reduction in proinflammatory (e.g., IL-1β, -6, NF-kB, and TNF-α) and oxidative (e.g., MDA and MPO) markers was noticed. In addition, hemin was also capable of increasing the expression of an anti-inflammatory cytokine, namely IL-10, and antioxidant (e.g., SOD and GSH) mediators. Therefore, regarding the preclinical evidence available about the pharmacological effect of hemin in several rodent models of disease, this drug may arise as a new potential approach in inflammatory-related human disorders, which is essential to evaluate it in a clinical setting.

## Figures and Tables

**Table 1:** Qualitative synthesis of the included studies.

Hemin-Related Parameters				Animal-Related Parameters					Biomarkers Assessed	Reference
Dose	Frequency	Route of Administration	Duration	Disease Animal Model	Specie	Strain	Gender	Age		
20 µmol/kg	3× week	SC	20 weeks	Emphysema	Rat	Wistar	Male	6 weeks	IL-8, IL-10, IL-17, Macrophages, MCP-1, MDA, MIP-2α, neutrophils, SOD, GSH, TNF-α	[18]
30 µmol/kg		IP	8 weeks	Liver fibrosis	Mice	C57BL/6		ND	HO-1, NFAT5, ROS, TGF-β1	[19]
				Steatohepatitis and fibrosis				8 weeks	HO-1, MCP-1, NF-kB, TGF-β1	[20]
	Single dose		-	LPS-stimulated inflammatory response	Rat	Sprague Dawley		10–11 weeks	HO-1, IL-1β, IL-6, LDH, MDA, SOD	[21]
						Wistar	Female			[22]
40 µmol/kg	Daily		8 days	Nephrotoxicity		Albino	Male	Adult	GSH, HO-1, IL-6, MDA	[23]
	Single dose		-	Acute liver failure		Sprague Dawley		6–7 weeks	HO-1, IL-1β, IL-6, MDA, MPO, NF-kB p65, Nrf2, TNF-α	[24]
		SC		Reproductive toxicity		Wistar		Adult	CAT, HO-1, GSH, iNOS, MDA, NO, TNF-α	[25]
	ND		30 days	Diabetes				ND	CAT, GSH, HO-1, MDA, MPO, SOD, TNF-α	[26]
75 µmol/kg	Daily	IP	2 days	Neutrophilic airway inflammation	Mice	BALB/c and DO11.10 transgenic	Female	8–10 weeks	HO-1, IL-6, IL-10, IL-17A, JAK1, JAK2, STAT3, T-cells	[27]
	Variable (days −2, −1, 12, 13, 21, 22, and 25)		27 days	Eosinophilic asthma		BALB/c	ND	ND	GATA3, HO-1, IL-4, IL-5, IL-17A, IL-17F, eosinophils, lymphocytes, macrophages, neutrophils, RORγt, SOCS3, STAT3	[28]
			27 days	Allergic airway inflammation		BALB/c, DO11.10, and Bas-TRECK transgenic		6–8 weeks	Basophils, eosinophils, IL-4, T-cells	[29]
80 µmol/kg	Single dose		-	Acute pulmonary inflammation		CD1 (WT and A2A gene-deficient) and C57BL/6 (WT and A2B gene-deficient)	Male	8–12 weeks	HO-1, IL-6, TNF-α	[30]
8 × 10^4^ µmol/kg				Acute lung injury		C57BL/6 (WT and HO-1^flox/flox^)			HO-1, IL-6, NF-kB p65, NF-kB p52, TNF-α	[31]
7.5 × 10^−2^ mg/kg				Acute pancreatitis	Rat	Sprague Dawley		ND	HO-1, IL-10, TNF-α	[32]
								6–7 weeks		[33]
0.1, 0.3, or 1.0 mg/kg		PI		Pleurisy		Wistar		ND	CAT, GPx, GST, IL-1β, iNOS, MCP-1, MPO, NOx, SOD, TNF-α	[34]
0.1, 0.3, 1, or 3 mg/kg	Daily	IV	3 days	Influenza pneumonia	Mice	BALB/c	Female	5–6 weeks	IFN-γ, IL-6, IL-10, HO-1, MCP-1, TNF-α	[35]
1, 3, or 10 mg/kg	Single dose	SC	-	Temporomandibular joint arthritis	Rat	Wistar	Male	ND	HO-1, IL-1β, MPO, TNF-α, White blood cells	[36]
5 mg/kg	Daily	IG	9 days	Chronic gastric ulcers				8–9 weeks	COX-2, HO-1, HO-2, IL-1β, iNOS, Nrf2, TNF-α	[37]
	Single dose	IP	-	Peritonitis	Mice	C57BL/6		6–8 weeks	IL-6, Leukocytes, macrophages, polymorphonuclear cells, TNF-α	[38]
				Renal ischemia–reperfusion injury			ND	8–12 weeks	HO-1, IL-1β, IL-6, Neutrophils, nitrotyrosine, peroxidase, TNF-α	[39]
5 or 10 mg/kg	Daily		14 days	Experimental colitis		CD-1	Female	6 weeks	IL-10, TNF-α	[8]
			4 days				Male	6–10 weeks	IL-1β, IL-10, MPO, TNF-α	[7]
8 mg/kg	Single dose		-	Sepsis	Rat	Sprague Dawley		8–10 weeks	Caspase-1, Caspase-11, GSDMD, HMGB1, IL-1β, IL-18, TNF-α	[40]
10 mg/kg				Sepsis	Mice	C57BL/6		ND	GDH, HO-1, IL-1β, IL-6, LDH, MDA, SOD2, TLR4	[41]
		ND		Renal inflammation and vasoconstrictor dysfunction	Rat	Wistar		10–13 weeks	HO-1, IL-1β, iNOS, NF-kB	[42]
10 or 30 mg/kg	Daily	IP	14 days	Neurotoxicity				ND	CAT, GSH, HO-1, IL-1β, TNF-α	[43]
20 mg/kg	Every 2 days		7 days	Experimental colitis	Mice	C57BL/6	Female	6–8 weeks	4-HNE, IFN-γ, IL-1β, IL-6, IL-12, iNOS, Ly6C, macrophages, MDA, MPO, NO, NF-kB p-p65, STAT1, STAT6, TNF-α	[44]
			14 days	Renal failure	Rat	Wistar	Male	ND	4-HNE, GSH, GSH-Px, HO-1, MPO, SOD	[45]
25 mg/kg	Daily		5 days	Myocardial infarction				ND	eNOS, GSH, HO-1, LDH, MDA, TNF-α	[46]
	3× week		ND	Endometrial hyperplasia			Female	9–10 weeks	GSH, IL-1β, iNOS, MDA, NOx, SOD	[47]
	Every 2 days		6 days	Vascular dysfunction	Mice	C57BL/6	Male	6–12 weeks	3-nitrotyrosine, 4-HNE, eNOS, Hmox1, macrophages, HO-1, IL-6, ROS	[48]
30 mg/kg			30 days	Penetrating keratoplasty	Rat	Wistar	Female	6–8 weeks	HO-1, iNOS, macrophages, MCP-1, MIP-1α, TNF-α	[49]
	2× week		8 weeks	Obesity		Zucker fatty and Zucker lean	Male	12 weeks	8-isoprostane, HO-1, IL-1β, IL-6, macrophages, nitrotyrosine, TGF-β1/2/3, TNF-α	[50]
	Single dose		-	Acute lung injury	Mice	BALB/c	Female	ND	Caspase-1, IL-1β, MDA, MPO, HO-1, ROS	[51]
					Rat	Sprague Dawley	Male		HO-1, IL-6, MDA, MPO, NF-kB, Nrf2, SOD, TNF-α	[52]
				Acute pancreatitis				16 weeks	HO-1, MDA, NO, TNF-α	[53]
				Liver transplantation		Wistar		ND	HO-1, IL-6, MDA, NF-kB, Nrf2, SOD, TNF-α	[54]
		ND		Mastitis	Mice	BALB/c	Female	8–12 weeks	IL-1β, MPO, NLRP3, procaspase-1, ROS, TXNIP	[55]
40 mg/kg	2× week	IP	14 days	Rheumatoid arthritis	Rat	Sprague Dawley	Male	8–10 weeks	IFN-γ, IL-1β, IL-6, T-cells, TNF-α	[56]
40 or 90 mg/kg			28 days							
40 mg/kg + 120 mg/kg	2 daily adm + 1 adm	IP + SC	28 or 41 days							
50 mg/kg	Daily	IP	14 days	Hepatic toxicity			N.D.	1 week	GSH, HO-1, IL-1β, IL-6, MDA, NOx, STAT3, TNF-α	[57]
			14 days	Pulmonary toxicity		Wistar	Female	Adult	GSH, HO-1, IL-6, LDH, MDA, MPO, NF-kB, NO, SOD	[58]
	Every 2 days		21 days	Restenosis		Sprague Dawley	Male	ND	CD45, HO-1, IL-1β, IL-6, MCP-1, NF-kB, TNF-α	[59]
	Single dose		-	Major burn model				Adult	HO-1, IL-1β, IL-6, MDA, NF-kB p65, TLR4, TNF-α	[60]
				Acute kidney injury		Sprague Dawley (WT and PINK1 knockout)		8 weeks	Caspase-1, HO-1, IL-1β, IL-6, MDA, NGAL, SOD, TNF-α	[61]
				Sepsis	Mice	C57BL/6			HO-1, IL-6, MDA, SOD, TNF-α	
0.5%	Daily	Topical	13 days	Diabetes	Rat	Wistar		Adult	IL-10, TNF-α	[62]
	2× day		19 days						CAT, GPx, GSH, HO-1, IL-10, MDA, SOD, TNF-α	[63]
10%	N.D.		21 days			Sprague Dawley		ND	HO-1, IL-6, MDA, SOD, TNF-α	[64]

4-HNE, 4-hydroxynonenal; CAT, catalase; COX-2, cyclooxygenase-2; eNOS, endothelial nitric oxide synthase; GATA3, GATA3 binding protein; GDH, glutamate dehydrogenase; GPx, glutathione peroxidase; GSDMD, gasdermin D; GSH, glutathione; GSH-Px, glutathione peroxidase; GST, glutathione S-transferase; HMGB1, high-mobility group box 1; Hmox1, heme oxygenase 1 gene; HO, heme oxygenase; IFN-γ, interferon-γ; IG, intragastric; IL, interleukin; iNOS, inducible nitric oxide synthase; IP, intraperitoneal; IV, intravenous; JAK, janus kinase; LDH, lactate dehydrogenase; LPS, lipopolysaccharides; Ly6C, lymphocyte antigen 6 family member C; MCP-1, monocyte chemoattractant protein-1; MDA, malondialdehyde; MIP, macrophage inflammatory protein; MPO, myeloperoxidase; ND, not defined; NFAT5, nuclear factor of activated T-cells 5; NF-kB, nuclear factor kappa B; NLRP3, NOD-like receptor pyrin domain-containing protein 3; NO, nitric oxide; NOx, total nitrites; Nrf2, Nuclear factor erythroid 2 related factor 2; PI, pleural injection; RORγt, retinoic-acid-receptor-related orphan nuclear receptor gamma; ROS, reactive oxygen species; SC, subcutaneous; SOCS3, suppressor of cytokine signaling 3; SOD, superoxide dismutase; STAT, signal transducer and activator of transcription; TGF-β, transforming growth factor-β; TLR4, toll-like receptor 4; TNF-α, tumor necrosis factor-α; TXNIP, thioredoxin-interacting protein.

**Table 2:** Risk of bias assessment in the included studies.

Final Grade	References
High (15–20) points	[21,22,32,37,46]
Moderate (8–14) points	[7,8,18,19,20,23,24,25,26,27,28,29,30,31,33,34,35,36,38,39,40,41,42,43,44,45,47,48,49,50,51,52,53,54,55,56,57,58,59,60,61,62,63,64]

## Data Availability

There is no additional data created despite the information present in the manuscript and Supplementary Material. Nevertheless, further inquiries can be directed to the corresponding author.

## Supplementary Materials

The following supporting information can be downloaded at: https://www.mdpi.com/article/10.3390/biomedicines12040898/s1, Table S1: Application of the SYRCLE’s tool for assessing the risk of bias in each included study.
