# Supplementary material for: The Pharmacological Effect of Hemin in Inflammatory-Related Diseases: A Systematic Review
Source: Biomedicines. 2024 Apr 18;12(4):898. doi: 10.3390/biomedicines12040898 (PMC11048114; doi:10.3390/biomedicines12040898)
Supplement: Supplementary file 1 [file biomedicines-12-00898-s001.zip › biomedicines-2929850-supplementary.pdf]

Supplementary Material

Comprehensive search expression applied in the biomedical electronic bases MEDLINE (Pubmed), Web of Science and Scopus, including the results obtained:

MEDLINE (PubMed) - Research expression:

(((hemin[MeSH Terms]) OR (hemin[Title/Abstract])) AND ((inflammation[MeSH Terms]) OR (inflammat*[Title/Abstract])) AND ((disease models, animal[MeSH Terms]) OR ("animal model*") OR ("disease model*") OR ("animal experimentation*") OR ("nonclinical stud*") OR ("non-clinical stud*") OR ("preclinical stud*") OR ("pre-clinical stud*") OR ("in vivo") OR (rat*[Title/Abstract]) OR (mouse[Title/Abstract]) OR (mice[Title/Abstract]) OR (rodent*[Title/Abstract])))

Pubmed - Results: 272 results at March 7th 2023

Web of Science - Research expression:

(TI=(hemin)) OR (AB=(hemin)) AND (TI=(inflammat*)) OR (AB=(inflammat*)) AND (ALL=("animal model*")) OR (ALL=("disease model*")) OR (ALL=("animal experimentation*")) OR (ALL=("nonclinical stud*")) OR (ALL=("non-clinical stud*")) OR (ALL=("preclinical stud*")) OR (ALL=("pre-clinical stud*")) OR (ALL=("in vivo")) OR (TI=(rat*)) OR (AB=(rat*)) OR (TI=(mouse)) OR (AB=(mouse)) OR (TI=(mice)) OR (AB=(mice)) OR (TI=(rodent*)) OR (AB=(rodent*))

Web of science - Results: 283 results at March 7th 2023

Scopus - Research expression:

TITLE-ABS-KEY (hemin) AND TITLE-ABS-KEY (inflammat*) AND ALL ("animal model*") OR ALL ("disease model*") OR ALL ("animal experimentation*") OR ALL ("nonclinical stud*") OR ALL ("non-clinical stud*") OR ALL ("preclinical stud*") OR ALL ("pre-clinical stud*") OR ALL ("in vivo") OR TITLE-ABS-KEY (rat*) OR TITLE-ABS-KEY (mouse) OR TITLE-ABS-KEY (mice) OR TITLE-ABS-KEY (rodent*)

Scopus - Results: 555 results at March 7th 2023

Table S1. Application of the SYRCLE's tool for assessing the risk of bias in each included study.

Ref.	Sequence generation	Baseline characteristics	Allocation concealment	Random housing	Blinding (Performance bias)	Random outcome assessment	Blinding (Detection bias)	Incomplete outcome data	Selective outcome reporting	Other sources of bias	**Total**
37	1	2	2	2	2	2	2	2	2	2	18
21	1	2	2	2	0	2	0	2	2	2	15
22	1	2	2	2	0	2	0	2	2	2	15
32	0	1	2	2	0	2	2	2	2	2	15
46	0	1	2	2	0	2	2	2	2	2	15
42	0	2	2	2	2	1	0	1	2	2	14
53	0	2	2	2	0	2	0	2	2	2	14
18	0	2	2	2	0	1	2	1	2	2	14
47	0	2	2	0	0	2	1	2	2	2	13
33	0	2	2	2	0	2	1	2	1	1	13
59	0	1	2	2	0	2	0	2	2	2	13
52	0	1	2	2	0	2	0	2	2	2	13
25	0	1	2	2	0	2	0	2	2	2	13
50	1	2	2	2	0	1	0	1	2	2	13
20	0	2	2	2	0	2	0	2	1	2	13
51	0	1	1	2	0	2	0	2	2	2	12
57	0	1	1	2	0	2	0	2	2	2	12
8	0	2	2	2	0	2	0	0	2	2	12
24	0	2	2	2	0	1	0	1	2	2	12
60	0	1	1	2	0	2	0	1	2	2	11
44	0	2	1	2	0	1	0	1	2	2	11
7	0	2	2	0	0	1	1	1	2	2	11
41	0	1	2	2	0	1	0	1	2	2	11
61	0	1	2	2	0	1	0	1	2	2	11
27	0	1	2	2	0	1	0	1	2	2	11
55	0	1	2	2	0	1	0	1	2	2	11
58	0	1	2	2	0	1	0	1	2	2	11

54	0	1	1	2	0	1	1	1	2	2	11
23	0	0	1	2	0	1	1	1	2	2	10
56	0	2	1	2	0	1	0	1	1	2	10
62	0	1	1	0	0	2	0	2	2	2	10
63	0	1	2	0	0	1	1	1	2	2	10
45	0	1	2	2	0	0	0	1	2	2	10
64	0	1	2	1	0	1	0	1	2	2	10
48	0	1	1	2	0	1	0	1	1	2	9
19	0	0	2	2	0	0	0	1	2	2	9
35	0	1	0	2	0	1	0	1	2	2	9
26	0	1	2	0	0	1	0	1	2	2	9
31	0	1	1	0	0	1	1	1	2	2	9
43	0	1	2	0	0	1	0	1	2	2	9
34	0	1	1	0	0	1	0	1	2	2	8
40	0	2	1	0	0	1	0	1	1	2	8
28	0	0	1	2	0	1	0	1	1	2	8
38	0	1	1	0	0	1	0	1	2	2	8
39	0	1	1	0	0	1	0	1	2	2	8
49	0	1	0	0	0	1	0	2	2	2	8
36	1	1	0	0	0	1	0	1	2	2	8
30	0	1	0	0	0	1	0	2	2	2	8
29	0	1	1	0	0	1	0	1	2	2	8
